# Axon collateralization and focal myelin dystrophy alter action potential propagation in multicompartment pyramidal neuron models

**DOI:** 10.1371/journal.pcbi.1013733

**Published:** 2025-12-15

**Authors:** Nilapratim Sengupta, Jennifer I. Luebke, Christina M. Weaver

**Affiliations:** 1 Department of Anatomy & Neurobiology, Boston University Chobanian & Avedisian School of Medicine, Boston, Massachusetts, United States of America; 2 Department of Mathematics and Statistics, Franklin & Marshall College, Lancaster, Pennsylvania, United States of America; IISER P: Indian Institute of Science Education Research Pune, INDIA

## Abstract

Action potentials (APs) propagate along axons to enable precise and rapid neuronal communication. Axon collaterals extend a given neuron’s range by distributing signals to multiple targets, but also increase its electrical demands. Myelination facilitates saltatory conduction, dramatically enhancing transmission speed and ensuring conduction fidelity over long distances. The interplay between myelin dystrophy and axonal arborization remains largely unexplored, despite their role in the impairment of AP conduction in brain circuits. Here we used cohorts of biophysically detailed, multicompartmental models of rhesus monkey dorsolateral prefrontal cortex (dlPFC) layer 3 pyramidal neurons to investigate how axon collateralization, myelin dystrophy, and other structural and biophysical perturbations affect AP conduction velocity (CV). We identified core diameter and branch point location, particularly its distance from the soma, as key structural determinants of CV. Under pathological conditions, branch points emerged as sites of heightened vulnerability, where even focal myelin damage markedly impaired conduction. The efficacy of remyelination to restore CV depended on the number of segments that replaced each demyelinated region. Effective length constant and focal input resistance largely predicted CV reductions caused by structural changes. In addition to demyelination, CV was highly sensitive to variations in axoplasmic and membrane resistivities, the formation of myelin balloons, and ion channel redistribution. These findings offer a mechanistic framework for understanding AP conduction in myelinated axons with complex topologies, while also pointing to potential factors underlying individual differences in vulnerability to cognitive impairments. This work thus provides an important building block for multiscale modeling of network and behavioral effects of aging, as well as axonopathies such as multiple sclerosis, Alzheimer’s disease, and traumatic brain injury.

## Author summary

Nerve cells called *neurons* communicate by sending electrical signals (*action potentials*) to downstream cells along their long, branching extensions known as axons. In healthy brains, many *axons* are wrapped in *myelin*, a protective coating that helps these signals travel quickly and reliably. However, myelin can become damaged in aging and neurological disorders like multiple sclerosis, Alzheimer’s disease, and traumatic brain injury. We used computer models of neurons to explore how signal transmission is affected when some or all of the myelin in localized axonal regions is lost. While such damage can slow down or even block the signal—especially near points where the axon branches—some recovery can occur when myelin is repaired, or certain proteins are redistributed along *axons*. Our findings help explain how communication between neurons breaks down in disease and provide important building blocks for future simulation studies of normal and pathological brain aging.

## Introduction

The temporal properties of axonal action potential (AP) conduction—velocity, rates, and patterns—are critical for the timing and fidelity of synaptic transmission and thus the entire cascade of neuronal communication. While often depicted simplistically as linear structures, most central nervous system axons are extensively branched. Such complex topology enables signal transmission to multiple targets, expanding the spatial dynamic range of a neuron’s influence within neuronal networks [1–3]. At the same time, branch points also introduce impedance mismatches and additional current demands, which can challenge the fidelity and speed of conduction [4–6].

Myelin dramatically facilitates AP transmission in many axons by enabling saltatory conduction [7–10]. Structural features such as axon diameter [11–15], the lengths of nodes and myelinated segments [16,17], and myelin thickness [18,19] play crucial roles in this process. Disruption of these properties through injury, disease, and/or degeneration can impair, slow, or even eliminate AP conduction and thus dramatically impact nervous system function [20–22]. Disorders including multiple sclerosis (MS), Alzheimer’s disease (AD), Parkinson’s disease (PD), Huntington’s disease (HD), and diffuse axonal injury (DAI) are all associated with extensive myelin dystrophy and axonal degeneration [23–27]. Myelin abnormalities also occur with normal, non-pathological aging [28,29]. While remyelination provides some post-demyelination recovery, replacing previously demyelinated regions with shorter and thinner myelinated segments or redundant myelin, it is often inadequate [30,31].

The small diameters and complex topology of myelinated axons make it difficult to empirically interrogate how pathological changes affect AP conduction. Computational modeling offers a robust approach to overcome these challenges, predicting biophysical effects of structural alterations and generating testable hypotheses about underlying mechanisms and interventions. *In silico* studies have examined how parameters including axon diameter, myelin thickness, node spacing and axonal swelling influence AP conduction in myelinated axons [32–38]. Some modeling studies explored the effects of altered myelin, including demyelination and remyelination [39–42]. While numerical and analytical approaches have offered insights into the mathematical underpinnings of axonal conduction [43–45], these studies primarily focused on unbranched axons. Computational research exploring how axon branching impacts conduction [46,47] has focused largely on unmyelinated axons. Further, some modeling studies have examined myelinated collaterals [48,49], but did not model interactions between branching and pathologies such as myelin dystrophy, ion channel redistribution, and myelin ballooning. Yet it is precisely this interplay of axon branching and focal myelin pathology that may produce the greatest vulnerability to conduction failure: branch points already impose electrical demands that could be exacerbated further by local structural abnormalities. Understanding these interactions is thus critical for identifying mechanisms of selective vulnerability in neurodegenerative diseases and aging.

To address these questions, we developed biophysically detailed, multicompartment models of pyramidal neurons with myelinated, branched axonal arbors. These models are based on layer 3 (L3) pyramidal neurons in the rhesus monkey dorsolateral prefrontal cortex (dlPFC). We chose the dlPFC due to its critical role in higher-order cognitive functions, such as working memory [50,51], and because dlPFC pyramidal neurons exhibit extensive collateralization with branches projecting to both local and long distance intrinsic as well as long distance extrinsic targets [52,53]. L3 neurons in the cortex are known to be heterogeneous in their myelination patterns [54,55]. Our modeling approach aimed to capture this heterogeneity to explore potential physiological and pathological scenarios relevant to both typical and disrupted axonal conduction. This is thus an ideal neuronal model for studying how dystrophy alters conduction in complex axonal architectures.

We found that axonal branch points significantly increased conduction challenges, particularly when myelin dystrophy occurred near these locations. Effective length constant and focal input resistance played critical roles in explaining variability of AP conduction velocity (CV) and propagation failures across models. Ion channel redistribution accompanying demyelination had variable effects: some patterns exacerbated conduction deficits while others partially mitigated them. These predictions provide mechanistic insights into how structural and biophysical perturbations underlie conduction impairments in diverse neurological conditions.

## Results

**Fig 1:**
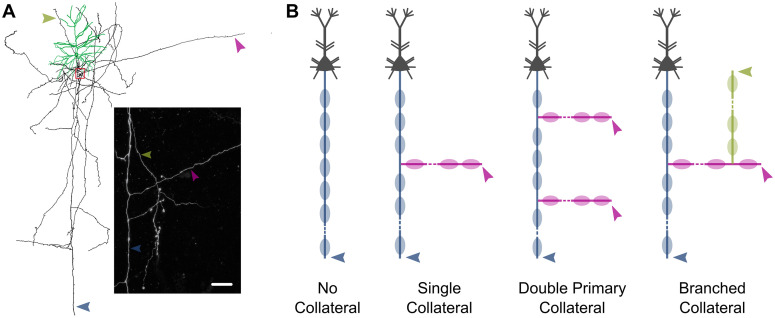
Model schematics. **(A)** Reconstruction of dendrites (green) and axonal arbor (black) of a representative rhesus monkey pyramidal neuron filled during *in vitro* whole cell patch-clamp recording. Inset showing higher magnification view of red boxed area (scale: 50µm). Arrows indicate different branches of the axonal arbor, from an *xy*-projection of the confocal microscopy image stack. **(B)** The four collateralization configurations explored in the present study. Arrows indicate respective locations for membrane potential recordings. Schematics are not to scale. In all panels, the main axon, primary collateral(s), and secondary collateral are indicated in blue, magenta, and olive green respectively.

In rhesus monkeys, dorsolateral prefrontal cortex (dlPFC) layer 3 (L3) pyramidal neurons exhibit complex branched axonal arbors (Fig 1A), including local and long-range collaterals. To represent this diversity for our *in silico* studies, we created four distinct axon models with progressively more complex branching patterns (Fig 1B). Each model included a main axon with 31 nodes and 30 myelinated segments. The configurations differed based on their collateralization: (1) the **No Collateral** model had an unbranched axon; (2) the **Single Collateral** model had one primary collateral branching from node 15; (3) the **Double Primary Collateral** model included proximal and distal collaterals emerging from nodes 10 and 20, respectively; and (4) the **Branched Collateral** model featured a primary collateral from node 15 of the main axon, and a secondary collateral branching from node 5 of the primary collateral. Primary collaterals had 11 nodes and 10 myelinated segments, and the secondary collateral had 6 nodes and 5 segments.

These schematics provide a structural framework for the simulations described in the following sections. Additional details on the biophysical implementation, parameter constraints, and model selection criteria are provided in the Methods section.

### Hierarchical branching and structural variations along collaterals influence CV

Previous studies have demonstrated that axon diameter, myelin thickness, and internodal length are critical determinants of CV [15,16,18,47]. To examine systematically how morphological changes along different pathways of the same axon may affect signal propagation, as a baseline before introducing dystrophy, we focused on the Double Primary Collateral and Branched Collateral models.

**Fig 2:**
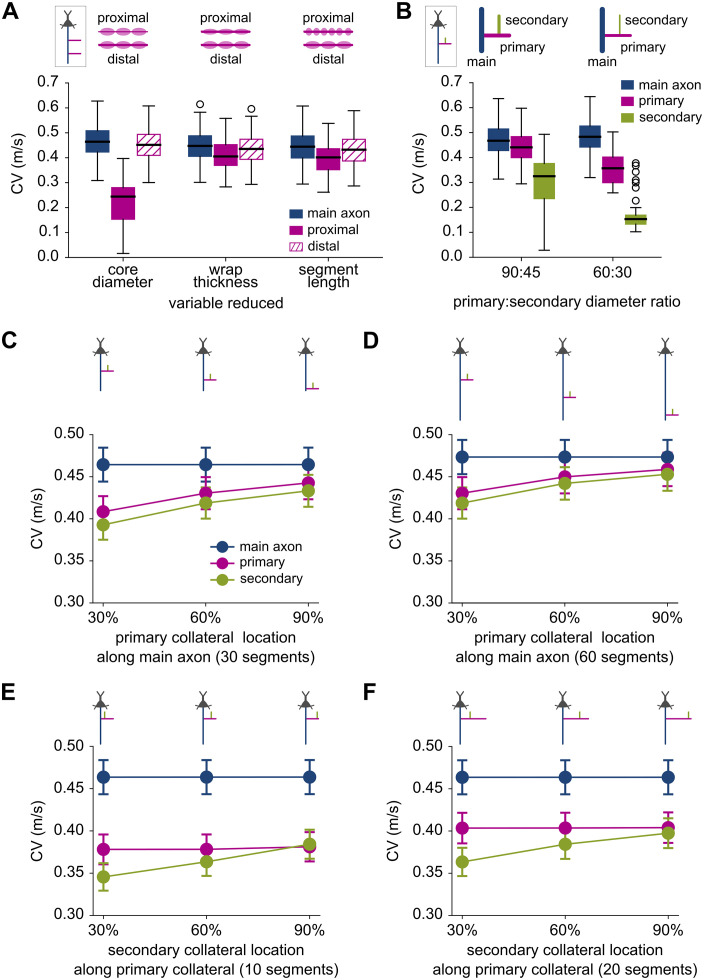
Hierarchical branching and structural variations along collaterals influence CV. **(A)** In the Double Primary Collateral model cohort, conduction velocity (CV) of action potentials (APs) along the main axon, proximal, and distal primary collaterals under three perturbations of the proximal primary collateral: reductions in core diameter, myelin wrap thickness, and myelinated segment length. **(B)** In the Branched Collateral model cohort, CV distributions along the main axon, primary and secondary collaterals under two conditions: moderately reduced diameter (90:45), and severely reduced diameter (60:30). **(C)** Mean CV as the primary collateral branch point moves along a 30-segment long main axon, occurring at either 30%, 60%, or 90% of the total main axon length. **(D)** Same as in panel C, but with a 60-segment long main axon, increasing the physical distance of the primary collateral origin from the soma. **(E)** Mean CV as the secondary collateral branch point moves along a 10-segment long primary collateral, at either 30%, 60%, and 90% of the total primary collateral length. **(F)** Same as in panel E, but with a 20-segment long primary collateral, increasing the physical distance of the secondary collateral origin from the soma. Cartoons above each panel illustrate the model analyzed and the corresponding perturbations. Error bars in panels C-F show mean ± SEM.

In the Double Primary Collateral model, we altered parameters in the proximal primary collateral by 50% in three separate ways: reducing core diameter, myelin wrap thickness, and myelinated segment length, relative to the unperturbed distal collateral. These structural alterations had differential effects on CV (Fig 2A). There was a strong interaction between pathway and condition (F_4,160_ = 139.90, p < 0.0001) with a large effect size (partial η^2^ = 0.778). These perturbations slowed CV significantly along the proximal collateral itself but did not affect CV in the main axon or distal collateral. Reducing axon diameter caused CV to slow the most (p < 0.0001); reducing myelin wrap thickness or myelinated segment lengths had similar, milder effects on CV.

Since we found that CV was much more sensitive to changes in axon diameter than myelin thickness or internode length, we next quantified how diameter reductions influenced CV in the Branched Collateral model (Fig 2B). We implemented two perturbation levels: in the moderate reduction (90:45), the diameters of the primary and secondary collaterals were set to 90% and 45% of the main axon’s diameter; in the severe reduction (60:30), the diameters were set to 60% and 30%, respectively. There was a significant interaction between pathway and condition (F_2,72_ = 54.84, p < 0.0001) with a large effect size (partial η^2^ = 0.604). CV along both collaterals slowed much more under the severe reduction (DR_60:30_) than the moderate reduction (DR_90:45;_ p < 0.0001); CV along the main axon was unaffected by these perturbations. The results highlight the biophysical impact of axon diameter on AP CV, particularly in collaterals which become progressively thinner as branching continues.

Finally, we analyzed how collateral branch point location affected signal propagation in the Branched Collateral model (Fig 2C-F). In these simulations, we systematically varied the origin of the primary or secondary collateral to 30%, 60%, or 90% along the length of their parent branch (main axon and primary collateral respectively). Across all cases, we observed that CV increased along the daughter branch(es) as the branch point moved farther from the soma. This trend was statistically significant for both the primary (p = 0.013; Fig 2C) and secondary (p = 0.002; Fig 2E) branch points, with medium effect sizes (standardized β = 0.201 and 0.256 respectively).

To evaluate whether these CV increases were driven primarily by the physical distance from the soma, or current reflection from the sealed ends of the axon and collaterals, we first repeated these simulations by doubling the length of the parent branches while keeping the physical branch locations unchanged (so that proportional locations were half of the originals; S1 Fig). The CVs were mostly identical to those in the original Branched Collateral model, except slightly higher along the respective branches whose lengths were increased (main axon, S1A Fig; primary collateral, S1B Fig). As a result, when the secondary branch point was located at 45% of the primary collateral length (black arrow, S1B Fig), the CV along the secondary collateral was then just slightly *below* that of the primary collateral, as we would expect. In fact, this outcome verified that the counterintuitive, opposite result in the original Branched Collateral model (Fig 2E) was caused by a numerical artifact: the primary collateral CV was computed from the penultimate node, which in this case coincided with the branch point and its corresponding current sink.

Next, we computed CV after doubling branch lengths of the Branched Collateral model while keeping relative branch locations unchanged (30%, 60%, 90%), so that branch points were now physically farther from the soma. In these cases, CVs along the daughter branch(es) were higher than for the original branch lengths (compare Fig 2 panels D vs. C, and F vs. E). CVs were also higher along the parent branches themselves (main axon in Fig 2D vs. Fig 2C; primary collateral in Fig 2F vs. Fig 2E), as the distal recording locations for CV calculations moved farther away from the soma with increased branch length. Together these simulations indicate that the observed CV increases were not due to distal current reflection. Rather, the soma acted as a proportionally greater current sink in shorter traversed axon paths, resulting in an overall CV increase from the axon initial segment to the branch points and recording locations that were farther away.

These findings illustrate some essential ways that collateral pathways and their branch locations shape CV dynamics, setting the stage for examining how myelin dystrophies affect signal propagation in branched axons.

### Focal demyelination near a branch point exacerbates CV slowdown

We recently showed that demyelination along entire unbranched axons slowed CV substantially, and that remyelination partially ameliorated this effect [39]. Here we considered dystrophy occurring in a limited region (contiguous zone comprising 20% of segments) of a collateralized axon: when such a region has lost either half or all of its myelin wraps (‘partial’ vs. ‘complete’ demyelination). We systematically repositioned this dystrophic zone along the main axon or primary collateral, irrespective of whether it spanned a branch point or an unbranched segment.

**Fig 3:**
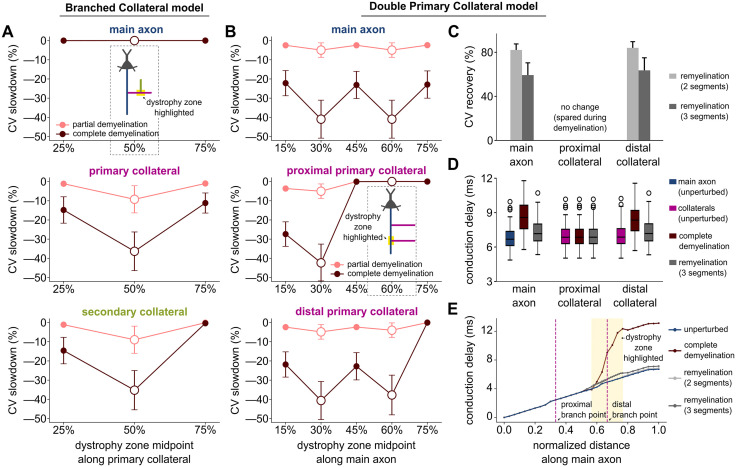
Focal demyelination near branch point exacerbates CV slowdown; remyelination strategies aid recovery with varying effectiveness. **(A)** CV slowdown after focal demyelination along the primary collateral (either partial or complete) of the Branched Collateral model. Panels (B-D): myelin perturbations in the Double Primary Collateral model: **(B)** CV slowdown after focal demyelination (either partial or complete) of the main axon. **(C)** CV recovery under remyelination with either two or three smaller and thinner segments, surrounding the distal branch point. **(D)** Mean AP conduction delays across the model cohort measured at 3 mm from the axon initial segment, along the main axon, proximal and distal collaterals respectively. Shown are the unperturbed, completely demyelinated, and remyelinated (with 3 new segments) conditions. **(E)** Conduction delay at each node relative to the first node of the main axon, measured in one representative model under the conditions from (D). Magenta dashed lines mark branch points where proximal and distal primary collaterals emerge. The dystrophy zone, highlighted in yellow, contains additional nodes in the remyelination conditions as shorter, thinner segments replace demyelinated ones. In panels A-B, data are shown along the different pathways; and *inset* model cartoons depict a representative location of the dystrophy zone (highlighted), aligned with the corresponding location along the horizontal axis.

In the Branched Collateral model, focal dystrophy in the primary collateral did not affect CV along the main axon (Fig 3A, *top*). However, this dystrophy slowed CV significantly in both the primary (χ² (5) = 223.33, p < 0.0001, Kendall’s W = 0.893) and secondary (χ² (5) = 231.25, p < 0.0001, Kendall’s W = 0.925) collaterals (Fig 3A, *middle* and *bottom*). CV slowdown was particularly severe when the dystrophic zone included the secondary branch point (schematic. Fig 3A: partial demyelination: W = 1251, r = 0.837 and W = 1268, r = 0.860 for primary and secondary respectively; complete demyelination: W = 1166, r = 0.721 and W = 1275, r = 0.870 for primary and secondary, all with p < 0.0001).

In the Double Primary Collateral model, demyelination along the main axon slowed CV significantly along all pathways (Fig 3B; main axon: χ² (9) = 398.43, p < 0.0001; proximal collateral: χ² (9) = 366.22, p < 0.0001; distal collateral: χ² (9) = 411.63, p < 0.0001; Kendall’s W = 0.886, 0.814, and 0.915 respectively). CV slowdown was much more pronounced when all myelin wraps were lost (burgundy lines) and when the dystrophic zone included a branch point (e.g., W = 1252, r = 0.838, p < 0.0001 for partial demyelination around proximal branch point; W = 1126, r = 0.666, p < 0.0001 for complete demyelination around distal branch point). Importantly, CV along the proximal collateral slowed significantly when dystrophy surrounded its branch point (W = 1275, p < 0.0001, r = 0.870), but was unchanged when the dystrophic zone was distal to its branch point. In the lower panel of Fig 3B, we were initially surprised to see that the distal collateral CV slowed more when the dystrophy was centered at the proximal collateral’s branch point rather than its own. As above, we found that this result was explained by the impact of the somatic current sink on the proximal branch point (S2 Fig).

Since dystrophy surrounding a branch point slowed CV the most, we examined how remyelinating a dystrophic zone with shorter and thinner internodes and associated new nodes affected AP propagation. When remyelinating a dystrophic zone surrounding the distal branch point, AP propagation improved along both the main axon and distal collateral (Fig 3C) but did not recover fully. The CV recovered less when the demyelinated segments were replaced by three shorter remyelinated segments rather than two (W = 1275, p < 0.0001, r = 0.870 for both). The proximal collateral pathway was unaffected by these demyelination and remyelination scenarios, as the dystrophic zone was downstream of the proximal branch.

For a different perspective, we interpreted these dystrophy-induced CV changes as time delays of AP propagation over a fixed distance (3 mm, Fig 3D). This revealed time delays of sufficient magnitude to disrupt precise communication with downstream targets. Focal demyelination surrounding the distal branch point did not change AP propagation along the proximal collateral (W = 97, p = 0.935, r = 0.645) compared to the unperturbed condition, but significantly delayed AP conduction along the main axon and distal collateral (W = 741, p < 0.0001, r = 0.871). These delays were partially mitigated by remyelination (W = 699, p < 0.0001, r = 0.772). Node-by-node analysis of conduction delays in one representative axon model highlights the cumulative impact of demyelination on AP propagation (Fig 3E). Under complete demyelination, CV slowed continuously throughout the dystrophic zone, resulting in a time delay of more than 6 ms relative to the unperturbed condition. Remyelination with shorter and thinner internode segments and new nodes reduced those time delays to less than 0.5 ms.

### Demyelination reduces the effective length constant of internodes

While the preceding analysis highlighted how focal demyelination and remyelination impact axonal signal propagation, the variability in CV changes across the model cohort warranted further investigation. In our previous work on myelin dystrophy in unbranched axon models [39], complex nonlinear interactions among many parameters made it difficult to isolate key parameters that slowed CV most. We realized that the effective length constant (λ_eff_; Fig 8B), quantifying the amount of passive signal attenuation within a myelinated segment, integrates these parameters in a functional way. As such, we explored whether changes in λ_eff_ might predict changes in CV.

**Fig 4:**
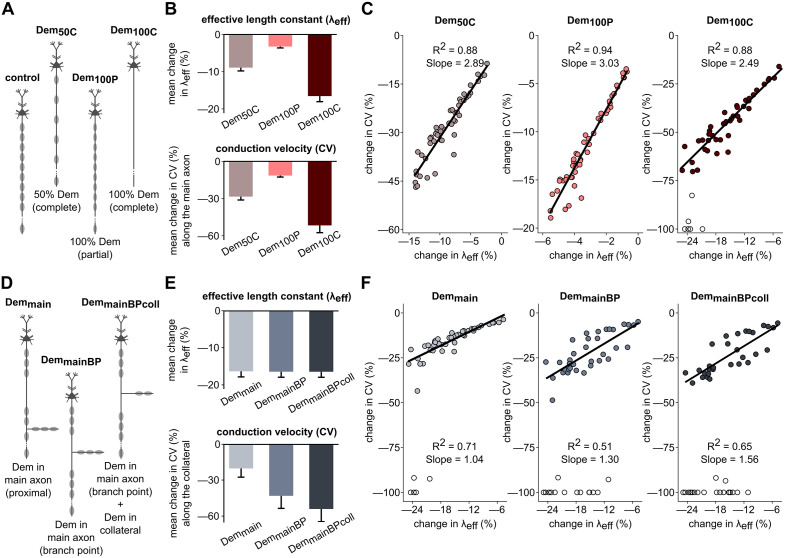
Changes to effective length constant during demyelination correlate with CV slowdown. **(A)** Schematics showing control (fully myelinated), partial and complete demyelination conditions for the No Collateral model: Dem_50C_, Dem_100P_, and Dem_100C_. **(B)** Mean percentage change in effective length constant (λ_eff_) and CV under each demyelination condition relative to the control case. **(C)** Percentage changes of CV versus λ_eff_ under demyelination conditions in A-B. **(D)** Schematics of demyelination conditions for the Single Collateral model: Dem_main_, Dem_mainBP_, and Dem_mainBPcoll_. **(E)** Mean percentage change in effective λ_eff_ and change in CV along the primary collateral under each demyelination condition, relative to the fully myelinated control. **(F)** Percentage changes of CV versus λ_eff_ under demyelination conditions in D-E. In panels C and F, we excluded models with any AP failures (*open circles*) from the regressions. See Methods for full description of perturbation conditions.

**Fig 5:**
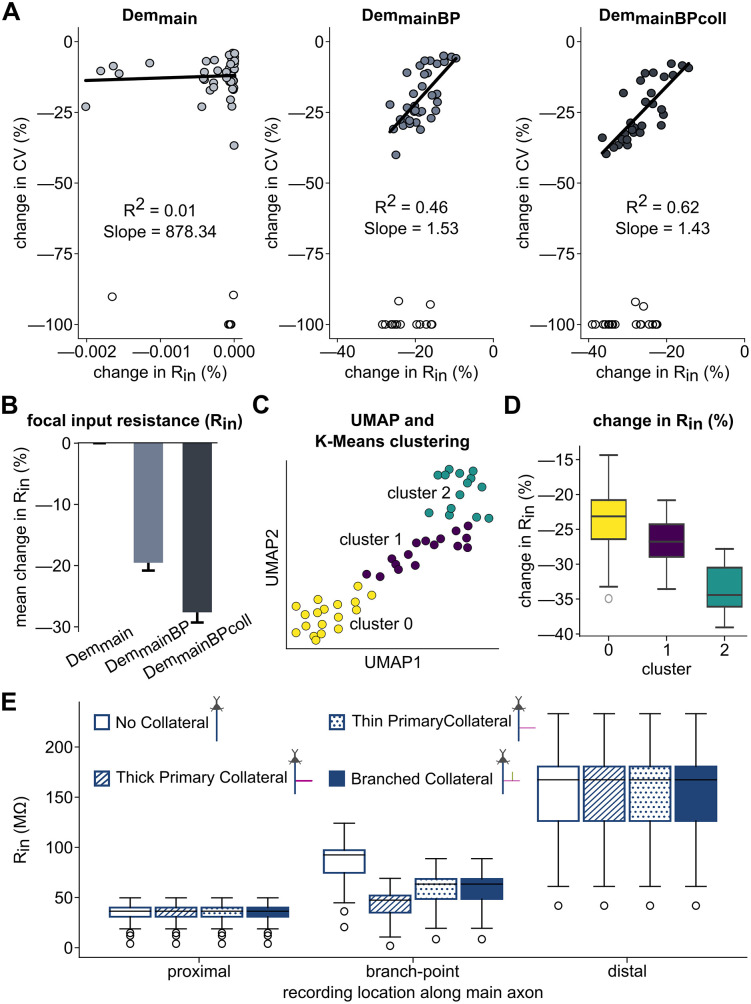
Focal input resistance varies with collateral configuration and correlates with CV slowdown. **(A)** Percentage changes of CV versus focal input resistance (R_in_) under the *Dem*_*main*_, *Dem*_*mainBP*_, and *Dem*_*mainBPcoll*_ demyelination conditions for the Single Collateral model, relative to the fully myelinated controls. Models with any AP failures (*open circles*) were excluded from the regressions. **(B)** Mean percentage changes in R_in_ at the branch point under the demyelination conditions. **(C)** CV change under the Dem_mainBPcoll_ condition projected onto the two dimensions of the UMAP transformation, colored by K-Means cluster membership. **(D)** Percentage change in R_in_ across the clusters identified in (C). **(E)** R_in_ measured at proximal, branch point, and distal locations along the main axon under different collateralization configurations: No Collateral, Thick Primary Collateral, Thin Primary Collateral, and Branched Collateral.

**Fig 6:**
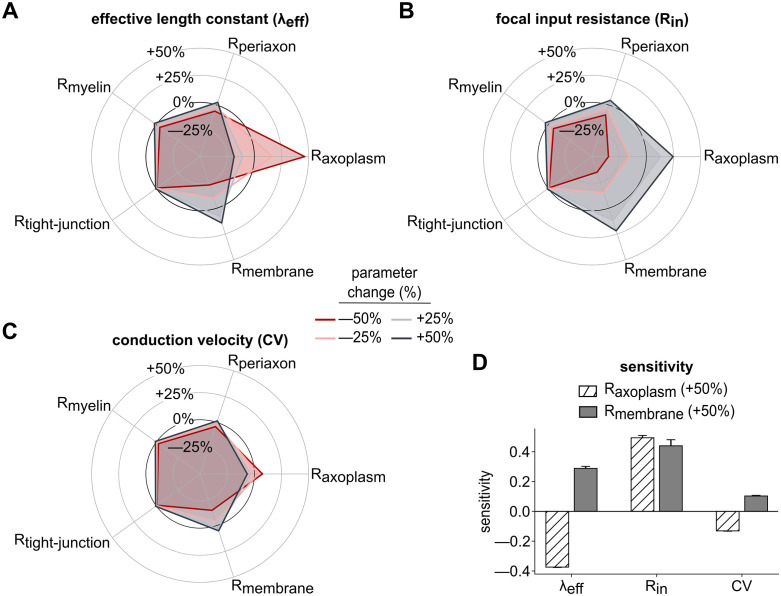
Effects of resistivity parameter variation on λ_eff_, R_in_, and CV in unbranched models. Radar plots showing the impact of changing resistivity parameters R_axoplasm_, R_periaxon_, R_myelin_, R_tight-junction_, and R_membrane_ on **(A)** λ_eff_, **(B)** R_in_, and **(C)** CV. Each plot illustrates mean percentage changes across 50 models under four levels of resistivity adjustment: -50% (red), -25% (light red), + 25% (light gray), and +50% (dark gray). Error bars were omitted for clearer visibility. **(D)** Sensitivity of λ_eff_, R_in_, and CV to 50% increase in R_axoplasm_ and R_membrane_.

**Fig 7:**
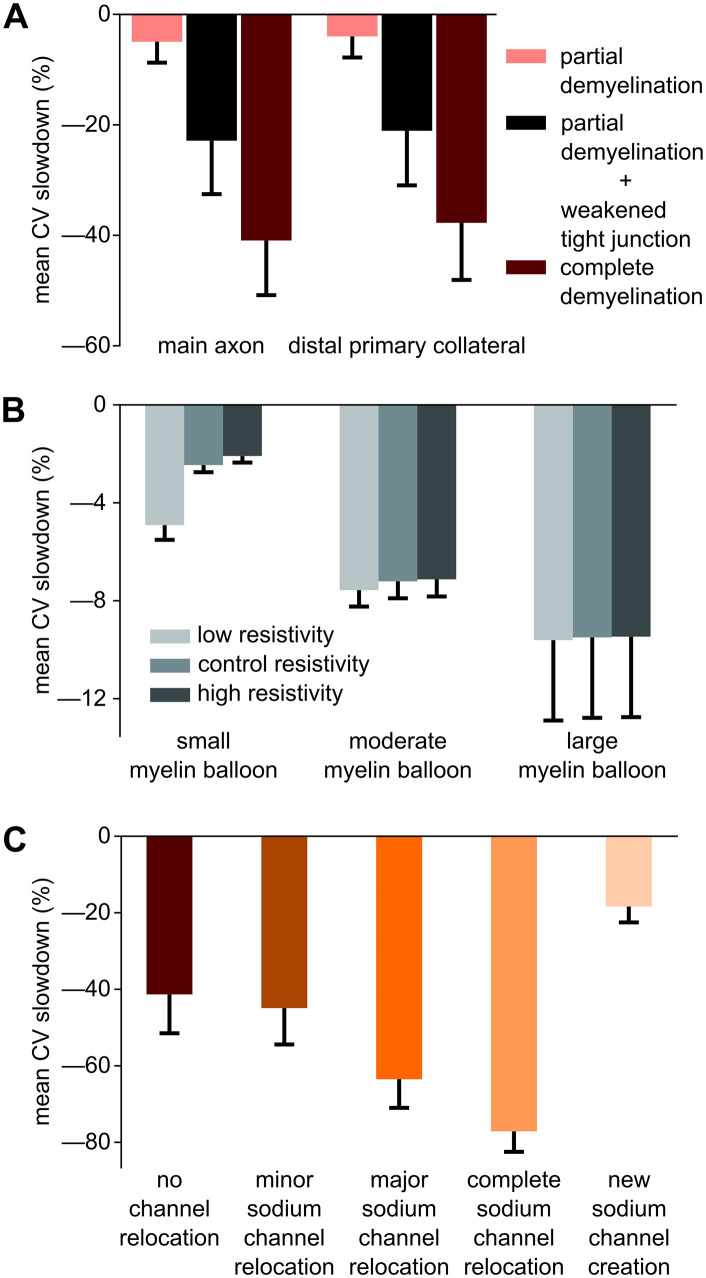
Simulated structural alterations and channel redistribution cause CV slowdown. **(A)** Mean CV slowdown along the main axon and distal primary collateral under partial demyelination, partial demyelination with weakened tight junctions, and complete demyelination. **(B)** Mean CV slowdown due to a myelin balloon formed halfway down the main axon, with varying internal resistivity levels (low, control, and high) and balloon sizes (small, moderate, large). **(C)** Mean CV slowdown along the main axon under complete demyelination, with simultaneous ion channel relocation/creation conditions in nodes and segments around the demyelinated zone (see Fig 8D).

**Fig 8:**
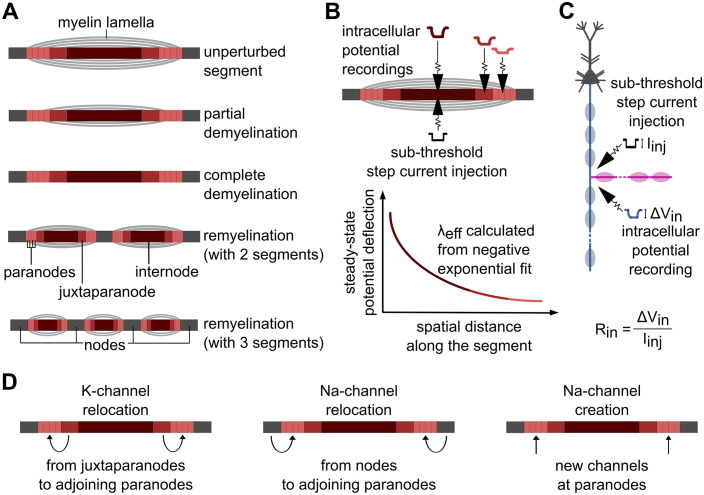
Model details and outcome measures. **(A)** Cartoon with a close-up view of myelinated segments (including paranodes, juxtaparanodes, and internodes) and adjacent nodes in the unperturbed model; under two demyelination conditions (partial and complete); and after remyelination with either two or three shorter, thinner segments. **(B)** Schematic depicting measurement of effective length constant (λ_eff_) within a myelinated segment. **(C)** Schematic depicting measurement of focal input resistance (R_in_). **(D)** Cartoons depicting ion channel relocation scenarios. In all cases, K^+^-channels moved from the juxtaparanodes to adjoining paranodes; at the same time Na^+^ channels were either relocated from nodes to adjoining paranodes, or newly created in the paranodes. Schematics are not to scale.

We first subjected our cohort of No Collateral models to three distinct demyelination scenarios (Fig 4A): Dem_50C_ (complete demyelination of 50% of segments), Dem_100P_ (partial demyelination of 100% of segments), and Dem_100C_ (complete demyelination of 100% of segments). Relative to the unperturbed models, both CV and λ_eff_ were significantly lower after all three demyelination scenarios (χ² (2) = 100, p < 0.0001, Kendall’s W = 1.000 for both CV and λ_eff_ for Dem_50C_, Dem_100P_, and Dem_100C_; Fig 4B). As expected, CV slowed the least for the mildest demyelination perturbation (Dem_100P_, removing half the myelin wraps from all segments), with CV slowing progressively more when removing all wraps from half or all myelinated segments respectively (Dem_50C_ and Dem_100C_). Strong linear relationships showed that CV and λ_eff_ decreased together significantly in all three demyelination scenarios (Fig 4C; Dem_50C_: CV = 2.89*λ_eff_ - 2.69, R² = 0.88; Dem_100P_: CV = 3.03*λ_eff_ – 1.61, R² = 0.94; Dem_100C_: CV = 2.49*λ_eff_ – 6.59, R² = 0.88; all p < 0.0001; standardized β = 0.940, 0.968, and 0.937 respectively).

Seeing that λ_eff_ was an excellent predictor of CV changes with dystrophy in unbranched models, we next subjected our cohort of branched, Single Collateral models (Fig 4D) to three focal demyelination patterns: Dem_main_ (20% of main axon segments demyelinated before the branch point), Dem_mainBP_ (demyelination surrounding the branch point), and Dem_mainBPcoll_ (demyelination around the branch point along both the main axon and collateral). The three localized demyelination conditions caused similar λ_eff_ reductions (F_2,147_ = 0.0058, p = 0.994, partial η^2^ = 0.000; Fig 4E, *top*). However, the amount of CV slowdown along the collateral differed significantly across conditions (χ² (2) = 83.24, p < 0.0001, Kendall’s W = 0.832), with the greatest CV slowdown occurring with Dem_mainBPcoll_, where demyelination surrounded the branch point in both the main axon and collateral (Fig 4E, *bottom*). As above, CV and λ_eff_ decreased together significantly in all demyelination scenarios (Dem_main_: CV = 1.04*λ_eff _+ 4.27, R² = 0.71, standardized β = 0.842; Dem_mainBP_: CV = 1.30*λ_eff _+ 0.51, R² = 0.51, standardized β = 0.711; and Dem_mainBPcoll_: CV = 1.56*λ_eff_ – 0.86, R² = 0.65, standardized β = 0.807; all p < 0.0001, Fig 4F). However, the lower R² values compared to those for unbranched axons suggest that additional factors beyond λ_eff_ might further help predict CV slowdown after demyelination.

### Dystrophy-induced CV slowdown correlates with focal input resistance change

While λ_eff_ quantifies subthreshold voltage attenuation within a myelinated segment, focal input resistance (R_in;_ Fig 8C) is a measure of a neuron’s capacity to influence local voltage changes and contribute to signal propagation. To probe the interplay between demyelination and collateral branching further, we assessed R_in_ at the branch point of the Single Collateral models under the three demyelination conditions (Fig 5A). As expected for Dem_main_, where demyelination along the main axon occurred before the branch point, changes in focal branch-point R_in_ were negligible and uncorrelated with CV slowdown in the collateral (R^2 ^= 0.01, p = 0.64, standardized β = 0.072). However, in the other two conditions, which affect the branch point directly, CV and focal R_in_ decreased together significantly (Dem_mainBP_: CV = 1.53*R_in_ + 8.73, R² = 0.46, standardized β = 0.678; and Dem_mainBPcoll_: CV = 1.43*R_in_ – 12.78, R² = 0.62, standardized β = 0.788; both p < 0.0001). Focal R_in_ changes were greater under Dem_mainBPcoll_ than Dem_mainBP_ (t-statistic = 40.78, p < 0.0001, Cohen’s d = 5.83; Fig 5B).

As a complementary, unsupervised approach to understanding variability across the Dem_mainBPcoll_ models, we employed Uniform Manifold Approximation and Projection (UMAP), a nonlinear dimensionality reduction technique increasingly used in neuroscience for extracting meaningful patterns from complex datasets [56,57]. UMAP embedding of the Dem_mainBPcoll_ condition separated the data along two nonlinear dimensions, and subsequent K-Means clustering assigned models to discrete clusters in the reduced parameter space (Fig 5C). We found that changes in R_in_, which was not included explicitly in the UMAP embedding, differed between the clusters (H(2) = 22.74, p < 0.0001, η^2^ = 0.441; Fig 5D), reinforcing that R_in_ change remains a meaningful differentiator across models.

Since focal R_in_ quantifies the effect of local biophysics on signal propagation, we used it to analyze different collateralization configurations (Fig 5E). Proximal R_in_ was low, and similar in all conditions (No Collateral, Thick Primary Collateral, Thin Primary Collateral, or Branched Collateral; H (3) = 0.021, p = 0.999, η^2^ = 0.000). Focal R_in_ increased significantly with distance from the soma in No Collateral models (H (2) = 113.06, p < 0.0001, η^2^ = 0.756), showing spatially distinct impedance characteristics even without collaterals. At the branch point of the collateralized models, focal R_in_ differed significantly (H (3) = 76.60, p < 0.0001, η^2^ = 0.376) and was lowest in the Thick Primary Collateral model with its greater current sink. Adding a secondary collateral (Branched Collateral) did not change R_in_ relative to the Thin Primary Collateral model. Distal R_in_ was significantly higher than proximal, given the location’s distance from current sinks like the soma and collateral branch(es), and was similar in all conditions (H (3) = 0.048, p = 0.997, η^2^ = 0.000).

### Resistivity of the axoplasm and membrane affect λ_eff_, focal R_in_, and CV most

Thus far, our perturbations have been restricted to morphologic features of the axon: changing core axon diameter, internode length, and removing myelin wraps. In addition to such morphologic changes, we wanted to explore possible dystrophic effects of resistivity changes—of axoplasm, periaxonal space, myelin, tight junctions, and the axonal membrane. Having established that λ_eff_ and R_in_ correlated strongly with CV, we measured these three quantities after perturbing resistive components along the axon of our simplest, No Collateral model cohort (Fig 6). Three of these resistive components (R_periaxon_, R_tight-junction_ and R_myelin_) had minimal impact on λ_eff_, R_in_ halfway along the axon, and CV. All three output measures were affected most by the other two parameters, R_axoplasm_ and R_membrane_. Computing sensitivity of λ_eff_, R_in_, and CV as the percentage change in each output arising from a 50% increase of each parameter [58], we found that increasing R_membrane_ caused all three of these outputs to increase too (i.e., all sensitivities to R_membrane_ were positive). This made sense, especially since λ_eff_ and R_in_ were positively correlated with CV in Figs 4C and 5A. Likewise, it made sense that sensitivity of λ_eff_ to R_axoplasm_ was negative, and in turn that sensitivity of CV to R_axoplasm_ was negative too. While the sensitivity of R_in_ and CV to R_axoplasm_ had opposite signs, this does not necessarily contradict our findings above. Increase in R_axoplasm_ essentially restricts axial current flow, which while hindering AP propagation along the axon (thus reducing CV) would contribute to increased input resistance measured focally at a given point.

### Concomitant structural alterations amplify CV slowdown with demyelination

Finally, to complement our analyses of myelin dystrophy, we explored how structural changes such as tight junction weakening, myelin balloon formation, and ion channel redistribution affected CV in our Double Primary Collateral model cohort. Such changes are often observed in aging and in pathological conditions involving myelinated axons.

Tight junction weakening compromises insulation within the myelin sheath, exacerbating conduction deficits [59]. While changing tight junction resistivity alone had little effect on CV, combining partial demyelination with weaker tight junctions further amplified the CV slowdown observed previously (Fig 7A). This coupled perturbation slowed CV less than when segments were fully demyelinated, but its effect was significantly larger than for partial demyelination alone (χ^2^(5) = 233.63, p < 0.0001, Kendall’s W = 0.935).

Myelin balloon formation, observed in aging-related myelin pathologies [60], induced moderate CV slowdown (Fig 7B), with the extent of reduction dependent on balloon size and internal resistivity (F_4,196_ = 154.25, p < 0.0001, partial η^2^ = 0.759). Larger balloons and those with lower internal resistivity slowed CV the most, with effect sizes that were between those for partial and complete demyelination of an axonal region of comparable size (compare Fig 7A nd 7B).

Disruptions to myelin integrity are often associated with changes in the spatial distribution of ion channels, including their relocation to paranodal and internodal regions [61,62]. Pairing several ion channel relocation scenarios with our complete demyelination perturbations led to significant differences in CV slowdown (χ² (4) **=** 161.36, p < 0.0001, Kendall’s W = 0.807; Fig 7C). Migration of Na^+^ channels from nodes into paranodes slowed CV proportionally to the percentage of channels moved; complete Na^+^-channel relocation into paranodes slowed CV the most. In contrast, adding new Na-channels in the paranodes, without reduction or relocation of those in the nodes, largely mitigated demyelination-induced CV delays.

## Discussion

Axonal signal propagation dynamics arise from a complex interplay of structural and biophysical factors [3,5,15,63]. This *in silico* study examined how focal axonal dystrophies—present in many pathological conditions—slow AP conduction, and suggests mechanisms that can mitigate such slowdown, in individual branches of collateralized model axons. To our knowledge, this is the first computational framework to explicitly integrate axonal branching with focal myelin pathology in biophysically detailed, spatially extended multicompartmental models. Two functional measures, the effective length constant (λ_eff_) within localized internodes and focal input resistance (R_in_) at nodes, largely predicted the nonlinear interactions between axonal morphology and electrical properties that shape CV. We constrained our cohort of 50 models by empirical data from the cortex of rhesus monkeys, an animal model sharing many features with humans [64,65]. The results enhance our understanding of local and long-range neuronal circuits in the mature brain.

Structural features such as diameter, myelination, and internodal lengths influence CV, excitability, and temporal fidelity [46,66]. We found that core diameter and the location of a branch point along its parent branch were the most important structural features affecting CV. These factors likely influence AP conduction along collaterals emerging from the main axon at varying distances from the soma, particularly within laminar architectures [67], and across stripe-like clusters of intrinsic collateral terminals within the modular organization of the prefrontal cortex [52].

Timing disruptions in neural circuits can lead to cognitive deficits [51,68–70]. A critical factor contributing to such disruption is dystrophic myelin, a hallmark of numerous neurological disorders. In Alzheimer’s disease (AD), early-stage white matter changes—including myelin thinning, oligodendrocyte dysfunction, and subtle conduction deficits—precede overt neuronal loss and likely impair conduction along the long-range association fibers crucial for cognitive integration [24,71,72]. Parkinson’s disease (PD), the second most common neurodegenerative disease after AD, has traditionally been considered primarily a gray matter disorder. However, it is increasingly recognized to involve myelin pathology as well [25]. In multiple sclerosis (MS), demyelination affects both long-range and local projection neurons, with focal lesions often occurring along collateralized axons, initially more prominent in white matter, but eventually leading to extensive, confluent demyelination in gray matter as well [21,73]. In Huntington’s disease (HD) demyelination and associated white matter abnormalities are observed even in pre-symptomatic individuals, with myelin breakdown and altered g-ratios emerging before the onset of overt motor or cognitive symptoms [26,74]. Traumatic brain injury (TBI) often leads to diffuse axonal injury (DAI), characterized by paranodal disruption, oligodendrocyte loss, and myelin degeneration that compromise axonal conduction and contribute to cognitive impairments [27]. Chronic post-traumatic demyelination may also elevate the risk of developing AD, as the spatial patterns of cortical myelin loss after TBI resemble those seen in early AD [75].

The shared feature of demyelination across these diverse neurological conditions underscores the broad applicability of our modeling results. In these diverse pathological conditions, a variety of specific (e.g., PD and HD) or diffuse neuronal populations (e.g., AD, TBI) undergo progressive dystrophic changes. While our models are fundamentally based on layer 3 pyramidal neurons that possess axons that collateralize locally and over long distances, they are generalizable to a broad range of principal neurons having branched axons throughout the central nervous system. Our prior work [39] explored how demyelination affects entire unbranched axons; here, focal demyelination in collateralized models revealed that branch points are particularly sensitive to myelin damage. Branch-point myelin dystrophy amplifies the inherent impedance mismatch [76] and current sink effects, which can lead to CV delays and AP failures [6]. Dystrophy primarily slowed CV in affected branches and their downstream pathways, with minimal effects on upstream pathways. As such, parallel pathways between local- and long-range targets are an important mechanism to preserve circuit functionality. Notably, AP failures were observed in some cases of complete demyelination, consistent with the absence of compensatory sodium channel redistribution in most of our models—an effect further supported by our simulations involving targeted ion channel redistribution.

Previous work showed that remyelination can restore AP conduction almost fully [39,40,77]. Extending this, we found that the extent of CV recovery was inversely related to the number of remyelinated segments replacing the demyelinated zone. This is particularly relevant for aging, where internode segments in aged subjects can be very small (~3 µm long, [31]), or in conditions like MS, where remyelination is often temporary [30].

To understand better how demyelination impacts CV, we measured changes in λ_eff_ and R_in_ under different demyelination scenarios. Our λ_eff_ computes the effective attenuation length within an individual myelinated segment, related to both the classical electrotonic length constant λ [78,79] and the electrotonic transform of log attenuation in dendritic arbors [80,81]. In unmyelinated axons, CV is modulated by λ-changes in regions of flare or taper, perturbing uniform cable properties [45]. Similarly here, myelin loss disrupted cable properties and reduced λ_eff_. However, current spread in adjacent segments also shaped λ_eff_ (S3 Fig), so that complete demyelination of all segments reduced λ_eff_ more than partial demyelination of half the segments. Combined with our finding that voltage attenuation in dendritic arbors decreased with aging in these same neurons [82], we predict that aging affects signal propagation in both the axon and dendrites in pyramidal neurons of rhesus dlPFC.

While λ_eff_ was an excellent predictor of CV slowdown in *unbranched* demyelinated axons, it had less predictive value in *collateralized* axons. Focal R_in_ further predicted CV changes along the collateral when the dystrophy zone included the branch point. This explanation was reinforced by unsupervised clustering after dimensionality reduction of the model parameter space: mean R_in_ changes differed significantly across the clusters. R_in_ is analogous to an impedance calculation under direct current; our findings from Coskren et al. [82] suggest that demyelination would attenuate high-frequency signals even more.

The λ_eff_ and R_in_ measures also help elucidate our findings in unperturbed models (Fig 2). Fig 5E shows that the collateral diameter influenced R_in_ near the branch point but not the distal axon. This explains why perturbing the proximal collateral had little effect on CV in the main axon or distal collateral (Fig 2A). Fig 5E also shows that R_in_ increased with distance from the soma. This is consistent with our findings that CV increased as the collateral branch point moved farther away from the somatic current sink (Figs 2C-F and S1). Further, we analyzed AP failures after diameter reductions in the Branched Collateral model (Fig 2B). The two factors that distinguished successful vs. failed AP propagation were λ_eff_ and the nodal Na^+^-channel scale factor (S4 Fig). This finding predicts that thin myelinated collaterals of healthy neurons have high values of both λ_eff_ and nodal Na^+^ channel densities.

To complement the myelin dystrophy simulations, we perturbed other parameters that may change during aging or neurodegeneration. CV, λ_eff_, and R_in_ were highly sensitive to axoplasm and membrane resistivity. These findings align with classical cable theory [16,83] and our past work [39]. This is relevant for pathologies such as AD, in which cytoskeletal disorganization and impairments in axoplasmic flow are well-documented and could contribute to cognitive impairments [72,84]. While weakening tight junction resistivity alone had little impact on CV, pairing it with demyelination exacerbated CV slowdown. Assessing tight junction impairment in conditions such as MS where remyelination is often transient or inadequate [30] would constrain such predictions further.

We ended with two perturbations known to occur with aging. First, we predict that the formation of myelin balloons [60] disrupts normal axonal conduction. While the impact of myelin balloons on CV slowdown depends on their size and internal resistivity, their effects may be similar in magnitude to moderate demyelination. Second, concomitant changes in the density of Na^+^ and K^+^ channels [61,62,85] might partially compensate demyelination-induced CV deficits. In particular, Na^+^-channel migration from nodes to paranodes made the CV slow *more*, while Na^+^-channel creation in paranodes made the CV slow *less*. To improve our model predictions, we need to understand both phenomena better.

Since collecting empirical data on axons is extremely challenging, computational studies like this one suggest which experiments would constrain future predictions best. For example, three-dimensional electron microscopy (3DEM) could examine axonal branching patterns, collateral diameters, and the frequency of myelinated and unmyelinated regions within individual neurons [86–88]. Also, 3DEM could help identify conditions under which myelin dystrophy occurs preferentially to individual neurons while sparing others, versus widespread dystrophy for neurons near, say, individual dysfunctional oligodendrocytes [89,90]. Combining multiplexed immunohistochemistry and confocal microscopy could assess the colocalization of Na^+^ and K^+^ channels and myelin basic protein along axon collaterals in neurons from healthy and diseased brains [91,92]. Multielectrode array recordings can enhance our understanding of axonal electrical properties under various conditions [11,93,94]. As technology continually evolves, empirical data to quantify these and important factors from our study will become increasingly available.

Even beyond uncertain empirical measurements, we acknowledge that our modeling study has limitations. Simplified collateral geometries do not capture the true variability of axonal branching. While our simulated cohort included a range of feasible morphologic and electrical parameters throughout the models, we generally assumed that these parameters were equal in different regions of the axonal arbor. To apply our findings to a broad range of pathological conditions, we did not model other concomitant changes, such as synapse loss and hyperexcitability observed with aging in vitro [95,96]. Such simultaneous changes might work together as homeostatic mechanisms partially compensating each other to help maintain function [69], or exacerbating one another, in a given context. Finally, we modeled this pyramidal neuron as electrically isolated from its neighbors. A recent study showed that modeling an axon as tightly packed among other myelinated fibers may increase AP conduction velocity and protect against failure [97].

Despite these limitations, the core mechanisms we uncover are likely to generalize across neuronal populations. Biophysically detailed multicompartment models have long served as adaptable platforms for studying diverse neural systems (reviews: [98,99]. The cohort of models we employed spans a wide parameter space that encompass most neocortical neurons, including interneurons. These properties enable our modeling framework to offer insight into AP conduction dynamics in arborized, myelinated axons under both healthy and pathological conditions where myelin integrity is compromised. The differential susceptibility of axons to varying degrees of myelin dystrophy may underlie the range of cognitive impairments observed across individuals. Incorporating these model neurons into microcircuit and large-scale network models [69,100,101] will provide an important and flexible test-bed for hypotheses spanning diverse spatial and temporal scales.

## Methods

### Pyramidal neuron and axon model

We extended our recent multicompartment model of a rhesus dlPFC L3 pyramidal neuron [39], constrained by morphologic and physiological data, creating three axon collateral configurations which complement our unbranched model (Fig 1B). Many details of the model were taken from our past work [39,102]. Briefly, the model included a soma, basal dendrites, and apical arbor, with passive membrane properties as well as eight active conductances (NaF, NaP, KDR, KA, KM, KAHP, CaL, AR). The main axon of the neuron model consisted of an axon hillock and an axon initial segment (AIS), having alternating nodes of Ranvier interspersed with myelinated segments [39,40,103]. At each branch point, the first node of a collateral was connected to a specific node along the main axon through axial resistances, with the geometry and biophysical properties of the connected sections governing current division across branches. No special mechanisms were introduced at these junctions; current flow followed Kirchhoff’s law, and action potential propagation emerged naturally from the model’s biophysical parameters. Each myelinated segment, along the main axon and collateral(s), was comprised of paranodal, juxtaparanodal, and internodal regions (Fig 8A), with tight junctions connecting the innermost myelin sheath to the axolemma. Axonal compartments had passive membrane properties throughout, with sodium and potassium channels focused in the nodes but also present at very low densities in the juxtaparanodes [40].

We implemented the models with the NEURON-Python interface [104,105], using a fixed time step of 0.025 ms. We simulated the in vitro current-clamp protocol from our past experiments [69,106,107] as in our previous modeling studies, clamping the somatic membrane potential at –70 mV and then applying 2-second current steps with the same total current injected empirically. We computed AP conduction velocity (CV) along any given branch using the time difference between APs recorded at the first node of the main axon (adjacent to the AIS) and the penultimate node along that branch (e.g., main axon, primary collateral, or secondary collateral). Thus, “CV along a branch” refers to the net propagation speed of an AP from its initiation near the soma to the distal end of a specified axonal branch and includes conduction through the main axon leading up to the collateral if applicable. We use this term throughout the manuscript to describe AP conduction to the terminal point of a given branch, rather than conduction restricted to a specific subregion (e.g., just within a collateral). The branch lengths are anatomically constrained and listed in Table 1, and thus both CV and conduction delay values inherently reflect these biological path lengths.

**Table 1:** Mean (± SD) lengths, core diameters and g-ratios across the model cohort for different axonal branches in the Branched Collateral models. Values are shown for models in which the primary and secondary collateral diameters were set to 90% and 75% of the main axon diameter, respectively—proportions used throughout the study unless otherwise specified.

	main axon	primary collateral	secondary collateral
**length**	2.72 ± 0.62 mm	0.90 ± 0.21 mm	0.45 ± 0.10 mm
**core diameter**	0.92 ± 0.07 µm	0.82 ± 0.06 µm	0.69 ± 0.05 µm
**g-ratio**	0.69 ± 0.07	0.66 ± 0.07	0.62 ± 0.07

### Control model cohort

We constructed the cohort of control models using Latin hypercube sampling [108] with 8 parameters: axon diameter, node length, myelinated segment length, number of myelin lamellae or wraps, myelin wrap thickness, and scale factors for the ratio of leak, sodium, and potassium conductances in the nodes compared to those in Scurfield and Latimer [40]. The chosen limits for these parameters, along with supporting empirical references, are detailed in our previous work [39]. We simulated 1600 of the Single Collateral models across the parameter space, and identified those that met these conditions: somatic firing rates of 13–16 Hz with +380 pA current step stimuli and silent without stimulation; conduction velocities 0.3–0.8 m/s; and proper saltatory conduction. Of the 74 models which met these selection criteria, 50 were chosen randomly to form the control. Table 1 summarizes the average lengths and g-ratios (ratio of axon diameter to total fiber diameter) along the different axonal pathways explored in the models. Although the modeled branch lengths do not replicate exact biological measurements, their relative proportions reflect the hierarchical organization of axonal projections in the rhesus monkey dlPFC. Long-range extrinsic (main axon), long-range intrinsic (primary collateral), and local intrinsic (secondary collateral) pathways target progressively shorter distances, with intrinsic projections typically spanning ~0.5 mm to a few millimeters [53,109].

### Localized output measures

To compute the effective length constant (λ_eff_) of a given myelinated segment, we injected a -80 pA current at its center and recorded the steady-state membrane potential deflection along the segment. Then, we fitted the spatial attenuation of the potential across half of the segment to a negative exponential to quantify the λ_eff_, selecting the value giving the minimum-residual fit (R² = 0.99 for a representative case; Fig 8B). We determined focal input resistance (R_in_) at a given location by injecting a -80 pA current and measuring the steady-state voltage response. R_in_ was calculated as the ratio of steady-state voltage deflection to injected current (Fig 8C). Our aim here was not to demonstrate that these measurements explicitly cause CV slowdown. Rather, we used λ_eff_ and R_in_ as integrative readouts, predicting how structural and biophysical heterogeneity across the model cohort contributed to the variability in CV changes under different perturbations.

### Simulating structural alterations

#### Structural variations along collaterals.

We perturbed the proximal primary collateral of the Double Primary Collateral model in three ways (Fig 2A): reducing core diameter, myelin wrap thickness, and myelinated segment length. In each perturbation, the parameter was reduced to half of its value in the unperturbed distal primary collateral. To perturb diameters in collaterals of the Branched Collateral model (Fig 2B), we reduced diameters of the primary and secondary collaterals relative to the main axon diameter. The notation X:Y indicates that the diameters of the primary and secondary collaterals were set to X% and Y% of the main axon diameter respectively. When modeling these structural variations, models which showed complete AP failure were excluded from subsequent analyses.

#### Demyelination and remyelination.

As in Ibañez, Sengupta et al. [39], we simulated both partial and complete demyelination of selected segments, where half or all myelin lamellae were removed. For focal demyelination, a consecutive sequence of 20% of myelinated segments were affected, in different portions of the axonal arbor. During remyelination, segments that originally had been demyelinated were replaced with either two or three shorter myelinated segments and new nodes between them. These shorter segments also had fewer myelin lamellae, equal to 75% of the unperturbed case.

Also, as in Ibañez, Sengupta et al. [39], the CV slowdown for demyelination conditions was computed relative to that of the unperturbed model. The CV recovery after remyelination conditions was computed as the percentage improvement in CV compared to the reduction observed in the completely demyelinated scenario. For one representative model, AP conduction delays were calculated for a propagation distance of 3 mm along each pathway (Fig 3D), under control (unperturbed), demyelination, and remyelination conditions.

For analyses including λ_eff_ and R_in_, we demyelinated the No Collateral model in three ways: Dem_50C_ (50% of all segments completely demyelinated, with every other segment affected), Dem_100P_ (all segments partially demyelinated), and Dem_100C_ (complete demyelination across all segments). We performed focal demyelination of the Single Collateral model in three ways: Dem_main_, where 20% of main axon segments were completely demyelinated, located before the collateral branch point; Dem_mainBP_, where 20% of main axon segments surrounding the branch point were demyelinated; and Dem_mainBPcoll_, where 20% of main axon segments surrounding the branch point and the first 20% of the primary collateral segments were demyelinated.

#### Resistivity parameter perturbations.

Across the 50-member cohort of the No Collateral model, we perturbed each resistivity parameter (for the axoplasm, periaxonal space, myelin lamellae, tight junctions, and membrane) one at a time by four scale factors (-50%, -25%, + 25% and +50%) in 20% of myelinated segments and adjoining nodes along the center of the axon. We computed the resulting mean percentage changes of λ_eff_, R_in_ and CV. Sensitivity of λ_eff_, R_in_ and CV in response to a 50% increase in each resistivity parameter was computed as the percentage change in the output variable, divided by 50%.

#### Other myelinated segment perturbations.

We used the Double Primary Collateral model to explore further structural and biophysical perturbations. To simulate weakening of tight junctions, we reduced tight junction resistivity by 90% [59] along selected demyelinated segments of the main axon around the distal branch point. To simulate balloon formation, we varied the diameter of the periaxonal space in the middle of the main axon between 2 µm (small balloon) and 20 µm (large balloon), consistent with the myelin balloon size range observed in aged monkey brains [60]. In some simulations we also increased or reduced internal resistivity by 200% and 80% respectively, representing the potential impacts of fluid composition of myelin balloon on AP conduction. Ion channel relocations (Fig 8D) were performed simultaneously for some demyelination studies. Relocations (Table 2) included the migration of potassium channels from juxtaparanodes to paranodes [61,62], and the heterogenous displacement as well as extended expression of sodium channels along the axons [85].

**Table 2:** Ion channel relocation perturbations modeled in conjunction with demyelination.

Condition	Sodium channels	Potassium channels
**no channel relocation**	no relocation	no relocation
**minor Na-channel relocation**	25% moved from nodes to paranodes	75% moved from juxtaparanodes to paranodes
**major Na-channel relocation**	75% moved from nodes to paranodes	75% moved from juxtaparanodes to paranodes
**complete Na-channel relocation**	100% moved from nodes to paranodes	75% moved from juxtaparanodes to paranodes
**new Na-channel creation**	extend across nodes and paranodes	75% moved from juxtaparanodes to paranodes

### Statistics

We used statistical analyses to evaluate the impact of structural and biophysical alterations on AP conduction across our model cohorts. We assessed normality using the Shapiro-Wilk test and applied non-parametric tests when the null hypothesis of normality was rejected. We used two-way repeated-measures ANOVA to examine the effects of conditions and pathways in models with multiple conditions, with significant pairwise differences being evaluated using Tukey HSD post hoc tests. For non-parametric analyses, we opted for the Friedman test, followed by Wilcoxon signed-rank or Kruskal-Wallis tests for group comparisons. Linear regression analyses were performed to assess correlations between λ_eff_, R_in_, and CV under various conditions. Paired t-tests were employed for specific comparisons where applicable. We used a significance level of α = 0.05, with a Bonferroni correction for multiple comparisons as appropriate. To aid interpretation of significant findings, we have reported corresponding effect size measures: partial eta squared (partial η^2^) for ANOVA, Kendall’s W for Friedman tests, eta squared (η^2^) for Kruskal-Wallis tests, rank-biserial correlation (r) for Wilcoxon signed-rank tests, Cohen’s *d* for paired *t*-tests and standardized β for regression analyses.

To explore changes in R_in_ across the cohort of models after demyelination, we employed an unsupervised analysis pipeline. We first applied Uniform Manifold Approximation and Projection (UMAP) for dimensionality reduction [110]. Input parameters for UMAP included a feature matrix consisting of the original 8 parameters used to construct the cohort, plus the changes in CV due to demyelination (UMAP hyperparameters: n_neighbors = 15, min_dist = 0.1, random_state = 42). UMAP reduced this parameter space down to two dimensions. We subsequently applied K-Means clustering (k = 3) to identify discrete clusters among the dimension-reduced parameter space [111]; this combination is becoming increasingly common to analyze complex datasets [112,113]. Kruskal-Wallis tests assessed significant differences in R_in_ changes between clusters.

Error bars shown in figures represent mean ± SEM. Significant differences are stated in the text, omitted visually, to maintain clarity in the figures.

## Supporting information

S1 Fig **(A)** CV along the main axon, primary and secondary collaterals of the Branched Collateral model when the main axon length was doubled (60 segments), but the physical location of the primary collateral branch point was kept the same as in Fig 2C. **(B)** CV along the main axon, primary and secondary collaterals of this model when the primary collateral length was doubled (20 segments), but the physical location of the secondary collateral branch point was kept the same as in Fig 2E.
(TIFF)

S2 Fig Mean change in CV in the Double Primary Collateral model, measured along the distal primary collateral when complete demyelination was focal and centered around either the proximal or distal branch point (original; as in Fig 3B bottom panel: 30% and 60% for dystrophy zone midpoint along main axon). In separate simulations, both branch points were shifted farther along the primary axon away from the soma, while maintaining the distance between them (‘shifted’, thick lines).
(TIFF)

S3 Fig Change in λ_eff_ measured along a demyelinated segment as more segments are demyelinated on each side. (TIFF)

S4 Fig Distribution of four key parameters (axon diameter, λ_eff_, R_in_ and nodal sodium channel conductance scale factor) in models that successfully propagated APs (working models) and those that failed (failed models), during diameter reduction explorations of the Branched Collateral Model shown in Fig 2B. Neither axon diameter nor focal R_in_ differed in working vs. failed models (U (49) = 190.5, p = 0.273 and U (49) = 189.0, p = 0.259 respectively). However, λ_eff_ and the nodal Na^+^-channel scale factor were significantly higher in working models vs. failed models (U (49) = 357.0, p = 0.010 and U (49) = 412.0, p = 0.0002 respectively).
(TIFF)
