# Supplementary figures and images for: Axon collateralization and focal myelin dystrophy alter action potential propagation in multicompartment pyramidal neuron models
Source: PLoS Comput Biol. 2025 Dec 15;21(12):e1013733. doi: 10.1371/journal.pcbi.1013733 (PMC12716699; doi:10.1371/journal.pcbi.1013733)

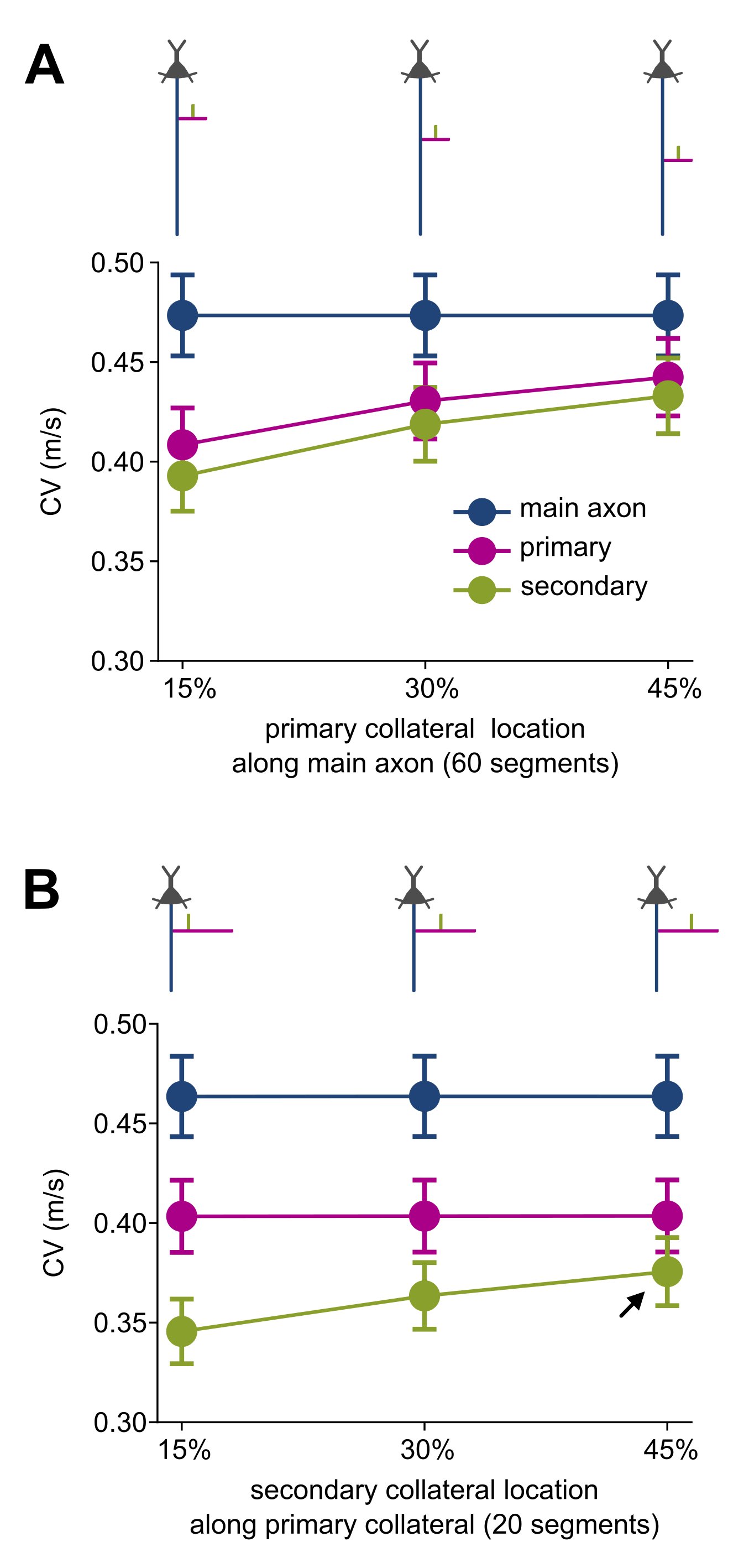

Supplement: S1 Fig — (A) CV along the main axon, primary and secondary collaterals of the Branched Collateral model when the main axon length was doubled (60 segments), but the physical location of the primary collateral branch point was kept the same as in Fig 2C. (B) CV along the main axon, primary and secondary collaterals of this model when the primary collateral length was doubled (20 segments), but the physical location of the secondary collateral branch point was kept the same as in Fig 2E. (TIFF) [file pcbi.1013733.s001.tiff]

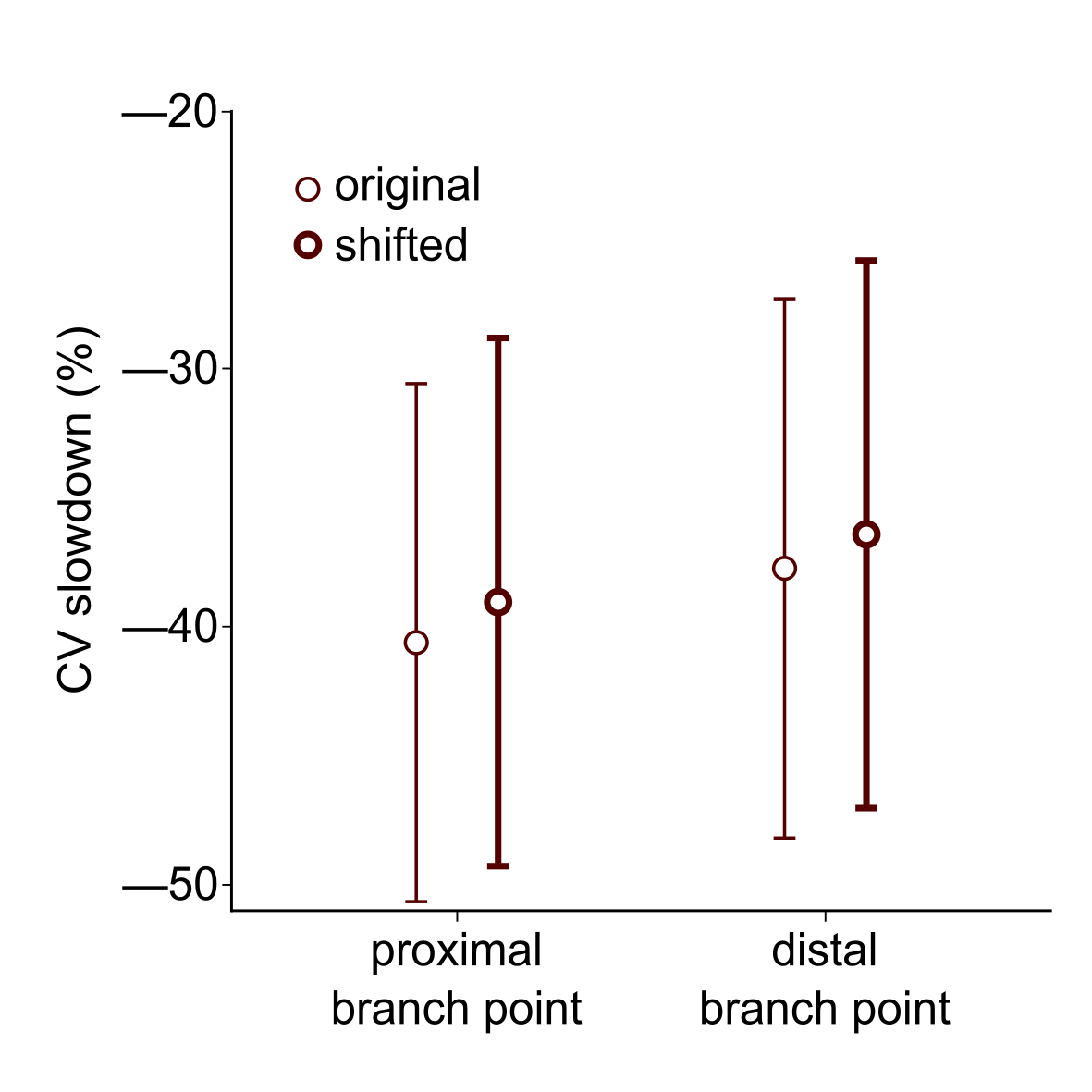

Supplement: S2 Fig — In separate simulations, both branch points were shifted farther along the primary axon away from the soma, while maintaining the distance between them (‘shifted’, thick lines). (TIFF) [file pcbi.1013733.s002.tiff]

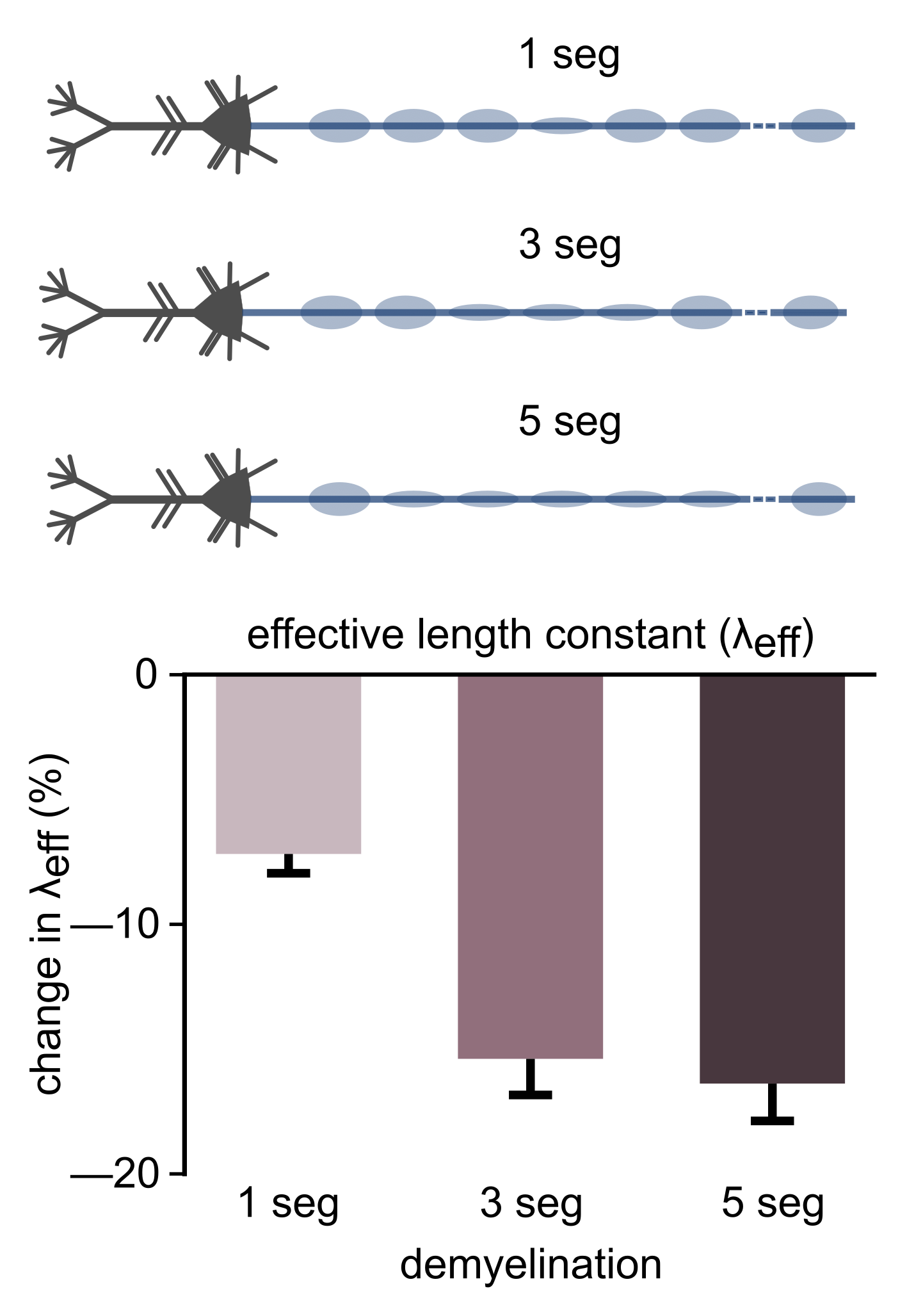

Supplement: S3 Fig — (TIFF) [file pcbi.1013733.s003.tiff]

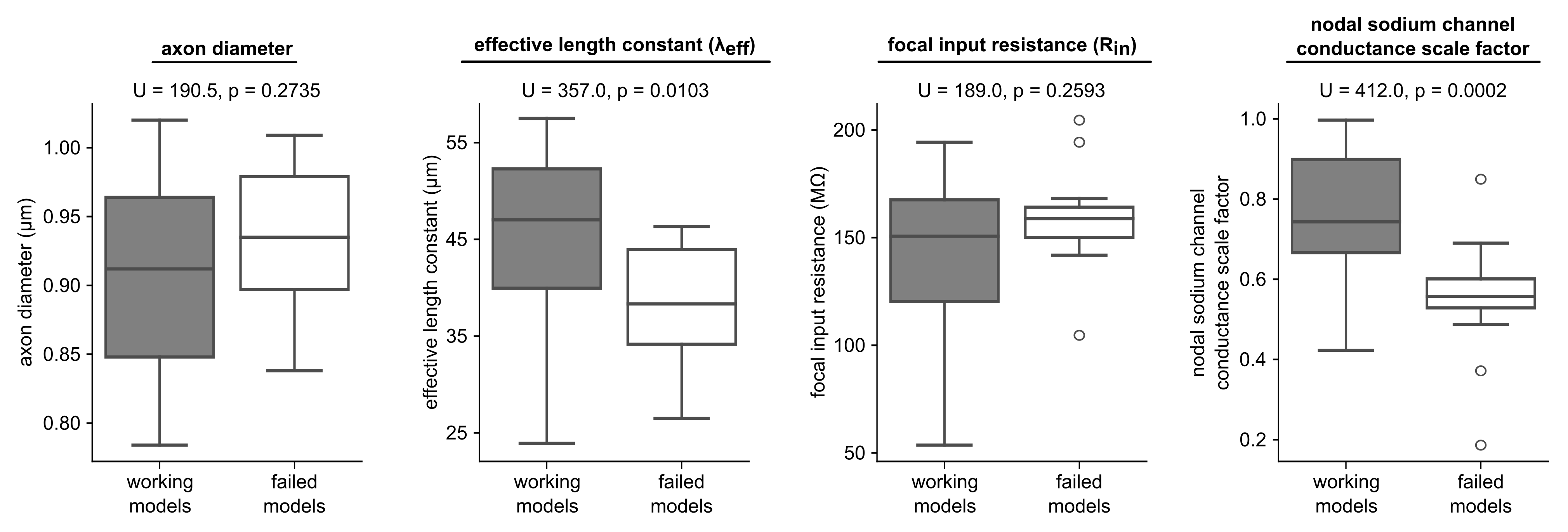

Supplement: S4 Fig — Neither axon diameter nor focal Rin differed in working vs. failed models (U (49) = 190.5, p = 0.273 and U (49) = 189.0, p = 0.259 respectively). However, λeff and the nodal Na+-channel scale factor were significantly higher in working models vs. failed models (U (49) = 357.0, p = 0.010 and U (49) = 412.0, p = 0.0002 respectively). (TIFF) [file pcbi.1013733.s004.tiff]
